# Supplementary material for: Self-Assembled BODIPY Nanoparticles for Near-Infrared Fluorescence Bioimaging
Source: Molecules. 2023 Mar 28;28(7):2997. doi: 10.3390/molecules28072997 (PMC10096313; doi:10.3390/molecules28072997)
Supplement: Supplementary file 1 [file molecules-28-02997-s001.zip › molecules-2229740-supplementary.pdf]

Supporting information

Self-Assembled BODIPY Nanoparticles for Near-Infrared Fluorescence Bioimaging

Jiale Wang [1], Zhao Jiang [1], Cheng Huang [2], Shimao Zhao [1], Senqiang Zhu [1,*], Rui Liu [1,*] and Hongjun Zhu [1]

1 School of Chemistry and Molecular Engineering, Nanjing Tech University, Nanjing 211816, China

2 Department of Chemical and Pharmaceutical Engineering, Southeast University ChengXian College, Nanjing 210088, China

* Correspondence: zhusenqiang1993@njtech.edu.cn (S.Z.); rui.liu@njtech.edu.cn (R.L.)

Synthesis and characterization of target compounds

All reagents were purchased from Sinopharm Chemical Reagent Co. Ltd and used as received unless otherwise mentioned. All the high-performance liquid chromatography (HPLC)-grade solvents used for spectroscopic study were without any further purification. Ultrapure water was used in the experiments and silica column chromatography was carried out on silica gel (200-300 mesh).

The complexes characterized by mass spectra, ^{13}C NMR and ^{1}H NMR. High resolution-mass (HR-MS) spectra were measured on an AB Sciex X-500B QTOF-mass spectrometer. ^{1}H NMR spectra and ^{13}C NMR spectra of the complexes in $CDCl_3$ or DMSO-d_6 solvent were measured on a Bruker Avance instrument, with tetramethylsilane for ^{1}H NMR spectra as an internal standard.

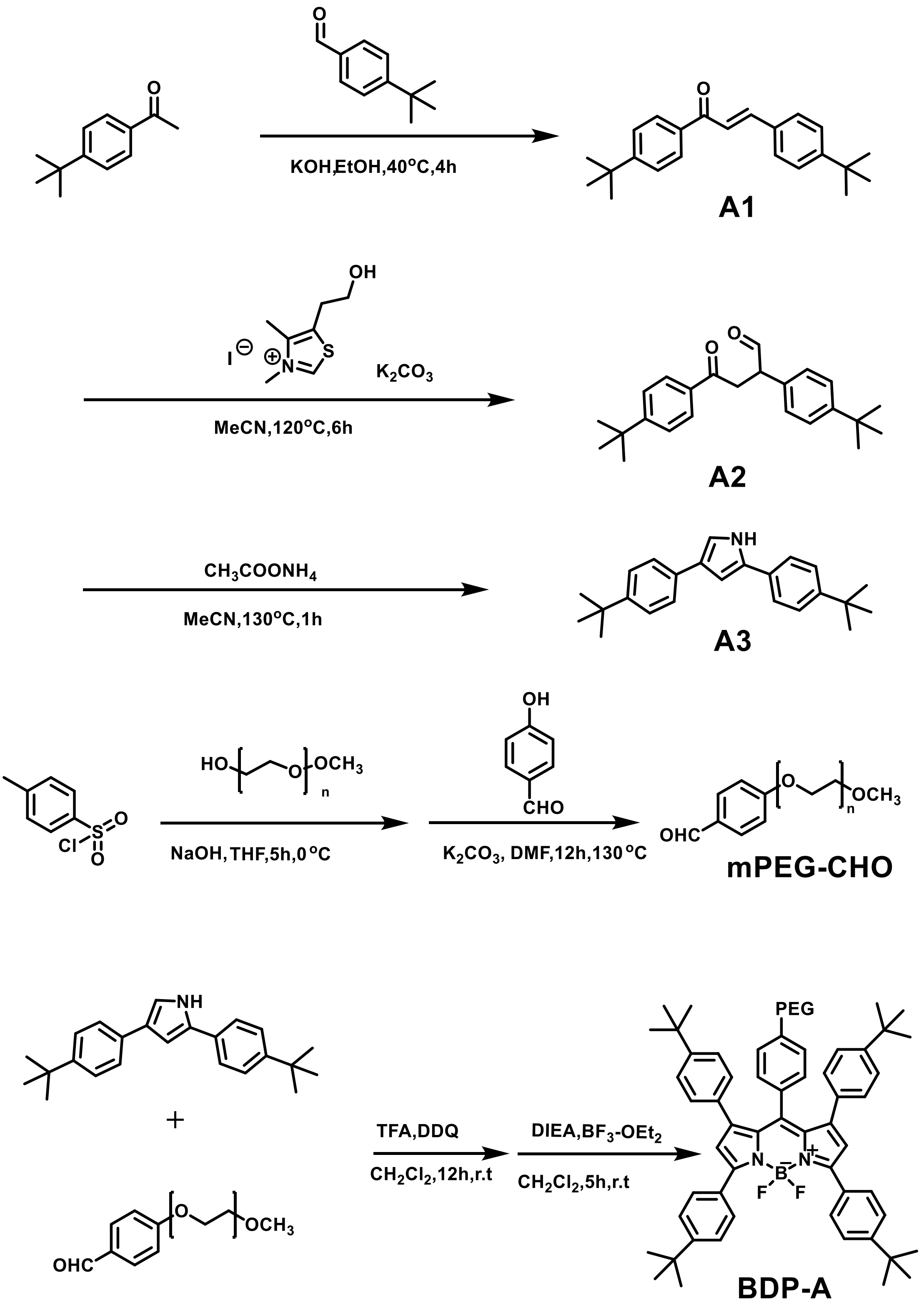

Scheme S1 Chemical structure and synthetic route of BDP-A

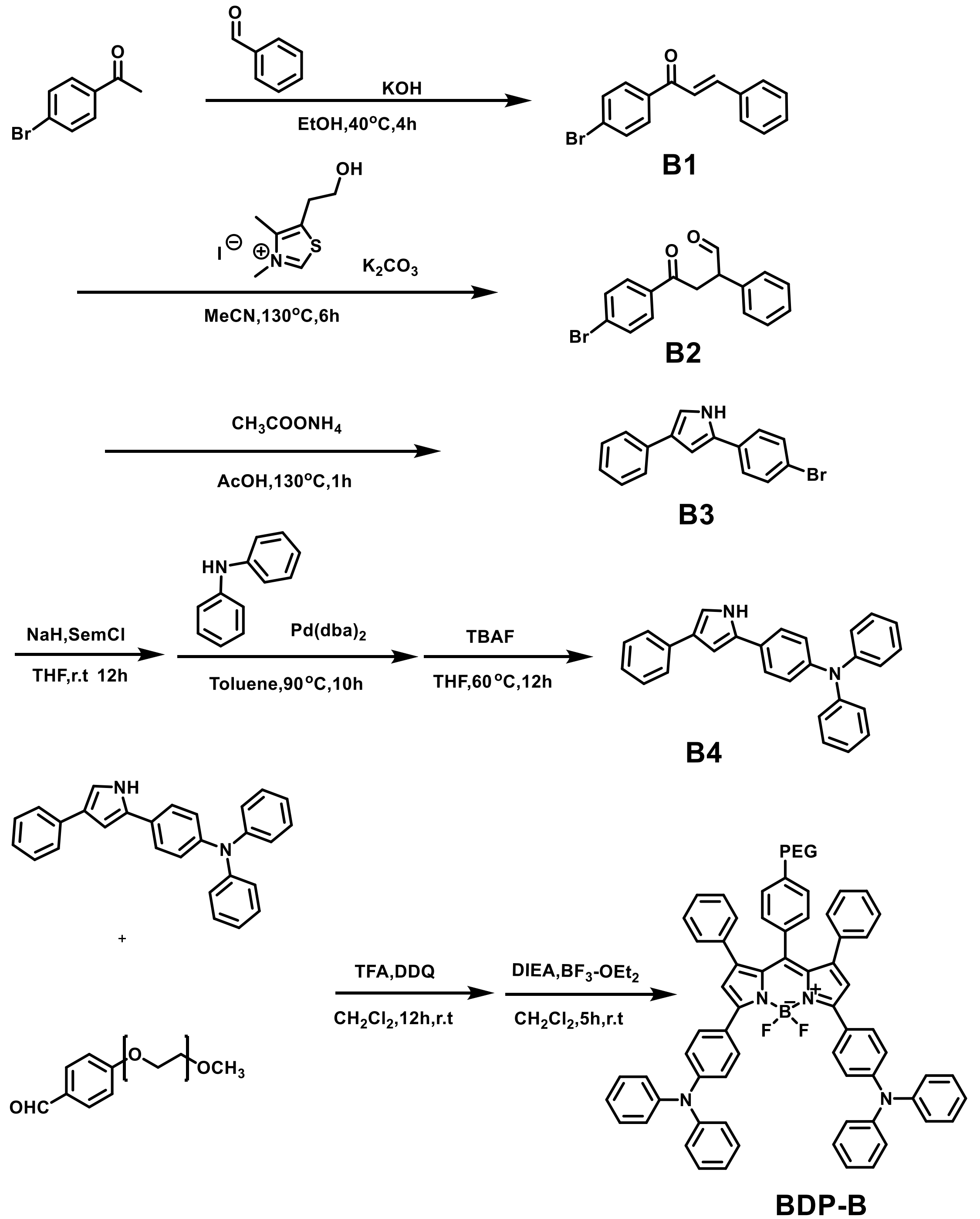

Scheme S2 Chemical structure and synthetic route of BDP-B

A1:4-tert-butylbenzaldehyde, 4-tert-butylacetophenone and 120 mL of ethanol were added into a two-mouth bottle, the reaction system is replaced with nitrogen and aqueous potassium hydroxide (20%) is added slowly dropwise to the bottle. Then the system was warmed up to 40 °C and the reaction was held for 4 h. After the reaction, the pale-yellow solid powder was obtained by filtration, and the resulting solid was recrystallized by dissolving with ethanol to obtain the pale yellow solid A1. Yield:78%. ^{1}H NMR (400 MHz, $CDCl_3$) δ 7.98 (*d, J* = 8.5 Hz, 2H), 7.84 – 7.78 (*m*, 1H), 7.59

(*t, J* = 7.8 Hz, 2H), 7.56 – 7.48 (*m*, 3H), 7.45 (*d, J* = 8.4 Hz, 2H), 1.36 (*dd, J* = 8.8, 5.0 Hz, 18H)

A2: A1, D-arabinose, NHC salt and potassium carbonate were added into a 250 mL two-necked flask, then acetonitrile (150 mL) was added and replace the reaction system with nitrogen, then warm up the system to 130 °C and reflux the reaction for 6 h. The progress of the reaction was tracked during the reaction using TLC. When the reaction was finished, the reaction solution was cooled to room temperature and the solvent was removed under vacuum, then 20 mL of ethyl acetate was added and the organic phase was extracted using 30 mL of deionized water × 3. The organic phase was dried using Na_2SO_4, and then the solvent was removed under vacuum. The crude product was purified using column chromatography V(PE): V(EA)=10:1; the final white solid A2 was obtained. Yield: 61%. ^{1}H NMR (400 MHz, $CDCl_3$) δ 9.80 (*s*, 1H), 7.92 (*d, J* = 8.5 Hz, 2H), 7.44 (*dd, J* = 26.0, 8.4 Hz, 4H), 7.20 (*d, J* = 8.3 Hz, 2H), 4.43 (*dd, J* = 8.3, 4.9 Hz, 1H), 3.92 (*dd, J* = 17.9, 8.4 Hz, 1H), 3.21 (*dd, J* = 17.9, 4.9 Hz, 1H), 1.33 (*d, J* = 7.8 Hz, 18H).

A3: A2 and ammonium acetate were added into a reaction flask, then acetic acid (60 mL) was added and dissolve, the system nitrogen replacement, and then the system was warmed to 130 °C reflux reaction for 1 h. After the reaction, the reaction solution was cooled to room temperature and filtered, and the solvent was removed under vacuum to obtain a yellow solid. The solid was dissolved using of ethyl acetate (30 mL), and then the organic phase was extracted with deionized water (30 mL × 3). The organic phase was dried using Na_2SO_4, and then the solvent was removed under vacuum. The resulting crude product was purified using column chromatography V(PE): V(EA) 8:1; a white solid A3 was obtained. Yield: 87%. ^{1}H NMR (400 MHz, $CDCl_3$) δ 8.38 (s, 1H), 7.50 (*d, J* = 8.3 Hz, 2H), 7.44 (*d, J* = 8.5 Hz, 2H), 7.42 – 7.36 (*m*, 4H), 7.07 (*s*, 1H), 6.77 (*d, J* = 2.1 Hz, 1H), 1.34 (*d, J* = 1.4 Hz, 18H). ^{13}C NMR (101 MHz, DMSO) δ 148.49, 147.71, 133.46, 133.15, 130.48, 125.88, 125.68, 125.02, 124.65, 123.68, 116.34, 103.10, 34.65, 34.54, 31.69, 31.60. HRMS m/z calculated for ($C_{24}H_{29}N$) $[M + H]^+$, 332.2334, found 332.2373.

B1: Benzaldehyde, p-bromoacetophenone and ethanol (120 mL) were added into a two-mouth bottle. The system was replaced with nitrogen, and the solution of potassium oxide (20%) was slowly dropped into the bottle. Then, the system was heated to 40 °C for 4 h. After the reaction, the light-yellow solid powder was extracted and filtered, and the resulting solid was dissolved in ethanol and recrystallized to obtain light yellow solid B1. Yield: 83%. ^{1}H NMR (400 MHz, DMSO) δ 8.11 (*d, J* = 8.6 Hz, 2H), 7.98 – 7.87 (*m*, 3H), 7.78 (*dd, J* = 12.0, 8.9 Hz, 3H), 7.51 – 7.44 (*m*, 3H).

B2: B1, D-arabinose, NHC salt and potassium carbonate were added into a 250 mL two-necked flask, then acetonitrile (150 mL) was added and the reaction system was replaced with nitrogen, then the system was heated to 130 °C and reflux the reaction for 6 h. The progress of the reaction was tracked during the reaction using TLC. When the reaction was finished, the reaction solution was cooled to room temperature and the solvent was removed under vacuum, then ethyl acetate (20 mL) was added and the organic phase was extracted using deionized water 30 mL× 3. The organic phase was dried using Na_2SO_4, and then the solvent was removed under vacuum. The crude product was purified using column chromatography V(PE): V(EA)=10:1; the final white solid B2 was obtained. Yield: 57%. ^1H NMR (400 MHz, $CDCl_3$) δ 9.81 (*s*, 1H), 7.85 (*t, J* = 6.8 Hz, 2H), 7.61 (*t, J* = 7.3 Hz, 2H), 7.46 – 7.32 (*m*, 3H), 7.29 (*dd, J* = 5.3, 3.0 Hz, 2H), 4.47 (*dd, J* = 8.5, 4.8 Hz, 1H), 3.93 (*dd, J* = 18.0, 8.6 Hz, 1H), 3.18 (*dd, J* = 18.0, 4.7 Hz, 1H).

B3: B2 and ammonium acetate were added into a reaction flask, then acetic acid (60 mL) was added to the system, nitrogen replacement, then the system was heated to 130 °C and reflux for 1 h. After the reaction, the reaction solution was cooled to room temperature and filtered, and the solvent was removed under vacuum to obtain a yellow solid. The solid was dissolved with ethyl acetate (30 mL), and then the organic phase was extracted with deionized water (30 mL x 3). The organic phase was dried using Na_2SO_4 and then the solvent was removed under vacuum. The resulting crude product was purified using column chromatography V(PE): V(EA)=8:1; a white solid B3 was obtained. Yield: 76%. ^1H NMR (400 MHz, $CDCl_3$) δ 8.44 (*s*, 1H), 7.62 – 7.50 (*m*, 4H), 7.39 (*tt, J* = 18.0, 8.9 Hz, 4H), 7.23 (*dd, J* = 15.1, 7.8 Hz, 1H), 7.17 (*s*, 1H), 6.89 – 6.80 (*m*, 1H).

B4: B3, NaH was added to a 100 mL reaction flask and dissolved with anhydrous Tetrahydrofuran (THF), the system was replaced with nitrogen and stirred for 30 min under ice bath conditions, then SemCl was added to the reaction system and reacted overnight. After the reaction, ethyl acetate (40 mL) was added to the reaction solution and the organic phase was extracted with deionized water (50 mL × 2). The organic phase was dried using Na_2SO_4 and then the solvent was removed under vacuum. The resulting crude product was purified using column chromatography V (PE): V (EA) =20:1 to give a yellow oily liquid. The oily liquid was diluted with 100 mL of toluene, and then diphenylamine, $Pd(dba)_2$, tBuNa were added to the solution, and the reaction system was replaced with nitrogen and refluxed at 90 °C for 10 h. The reaction solution was cooled to room temperature at the end of the reaction, the solvent was removed under vacuum, the crude product

was dissolved using ethyl acetate (50 mL), and then extracted using deionized water (50 mL × 2). The resulting organic phase was dried using Na_2SO_4, and then the solvent was removed under vacuum. The resulting crude product was then dissolved using THF (100 mL) and TBAF was added into the solution. The reaction system was replaced with nitrogen and the reaction was carried out at 60 °C for 12 h. After the reaction, the reaction solution was cooled to room temperature and the solvent was removed under vacuum. 40 mL of ethyl acetate was added, and the organic phase was extracted using deionized water (40 mL × 2). The resulting crude product was purified using column chromatography V (PE): V(EA) =8:1; a light green solid B4 was obtained. Yield: 36%. ^{1}H NMR (400 MHz, DMSO) δ 11.37 (*s*, 1H), 7.61 (*t, J* = 7.6 Hz, 4H), 7.36 – 7.26 (*m*, 7H), 7.12 (*t, J* = 7.3 Hz, 1H), 7.08 – 6.97 (*m*, 8H), 6.87 (*d, J* = 1.8 Hz, 1H). HR-MS m/z calculated for ($C_{28}H_{22}N_2$) [M + H]$^+$, 387.1817, found 387.1855. ^{13}C NMR (101 MHz, DMSO) δ 147.66, 145.37, 136.23, 132.56, 129.97, 129.00, 128.18, 125.45, 125.12, 124.84, 124.70, 124.00, 123.23, 116.82, 103.10.

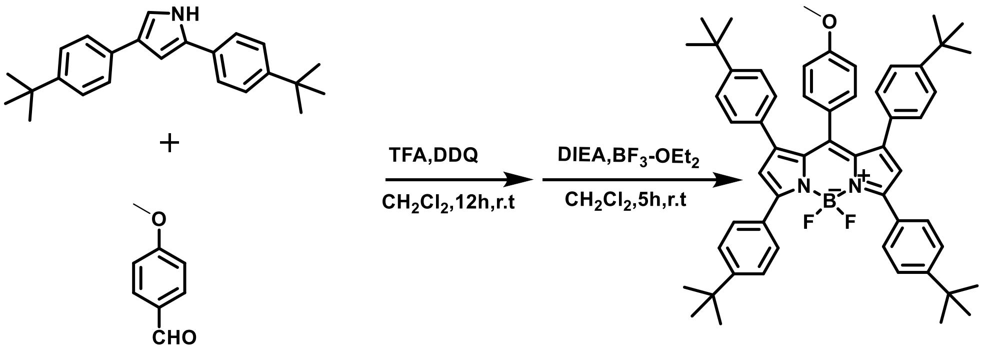

Scheme S3 Chemical structure and synthetic route of BDP-A with unattached PEG chains

BDP-A with unattached PEG chains: The mixture of A3 and 4-methoxybenzaldehyde was dissolved in anhydrous dichloromethane (50 mL) and one drop of trifluoroacetic acid was added to initiate the reaction. The reaction system was reacting in an ice bath under light-proof conditions for 12 h. Then DDQ was added to the reaction system and the reaction was continued for 5 h. After the reaction, the reaction solution was extracted using saturated aqueous sodium bicarbonate solution (50 mL × 3). The resulting organic phase was dried using Na_2SO_4, and then the solvent was removed under vacuum to give a purple solid. The resulting solid was dissolved using 50 mL of dichloromethane, 0.8 mL of DIEA was added and stirred for 30 min, and then 1 mL of $BF_3{\cdot}Et_2O$ was slowly added to the reaction system, at which time the reaction solution gradually changed from purple to dark red. The reaction was carried out at room temperature for 5h. After the reaction, the reaction solution was extracted with deionized water (50 mL×2), and the resulting organic phase

was dried with Na_2SO_4, and then the solvent was removed under vacuum. The resulting crude product was purified by column chromatography V (PE): V (DCM) =5:1 to give a red solid BDP-A with unattached PEG chains. Yield: 32%. ^{1}H NMR (400 MHz, CDCl3) δ 7.88 (*d, J* = 8.5 Hz, 4H), 7.49 (*d, J* = 8.5 Hz, 4H), 6.92 (*d, J* = 8.3 Hz, 4H), 6.73 (*dd, J* = 11.4, 8.5 Hz, 6H), 6.57 (*s*, 2H), 5.97 (*d, J* = 8.7 Hz, 2H), 3.54 (*s*, 3H), 1.37 (*d, J* = 6.5 Hz, 18H), 1.22 (*s*, 18H).

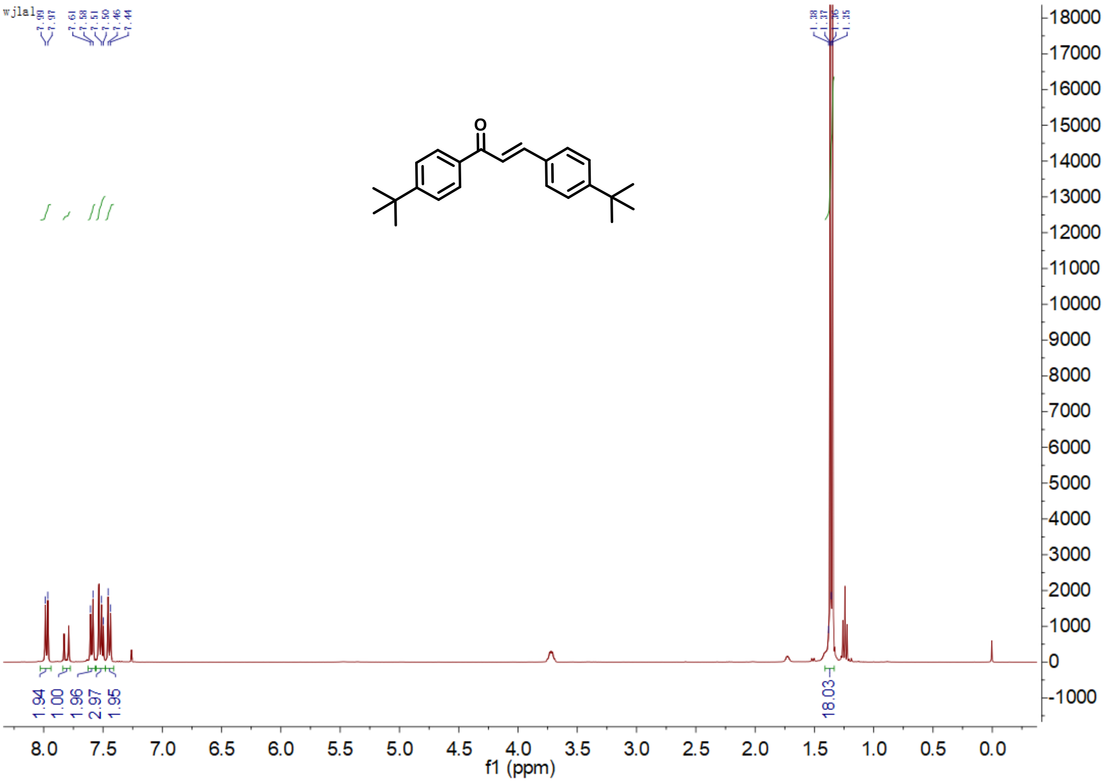

Figure S1. ^{1}H NMR spectrum of A1

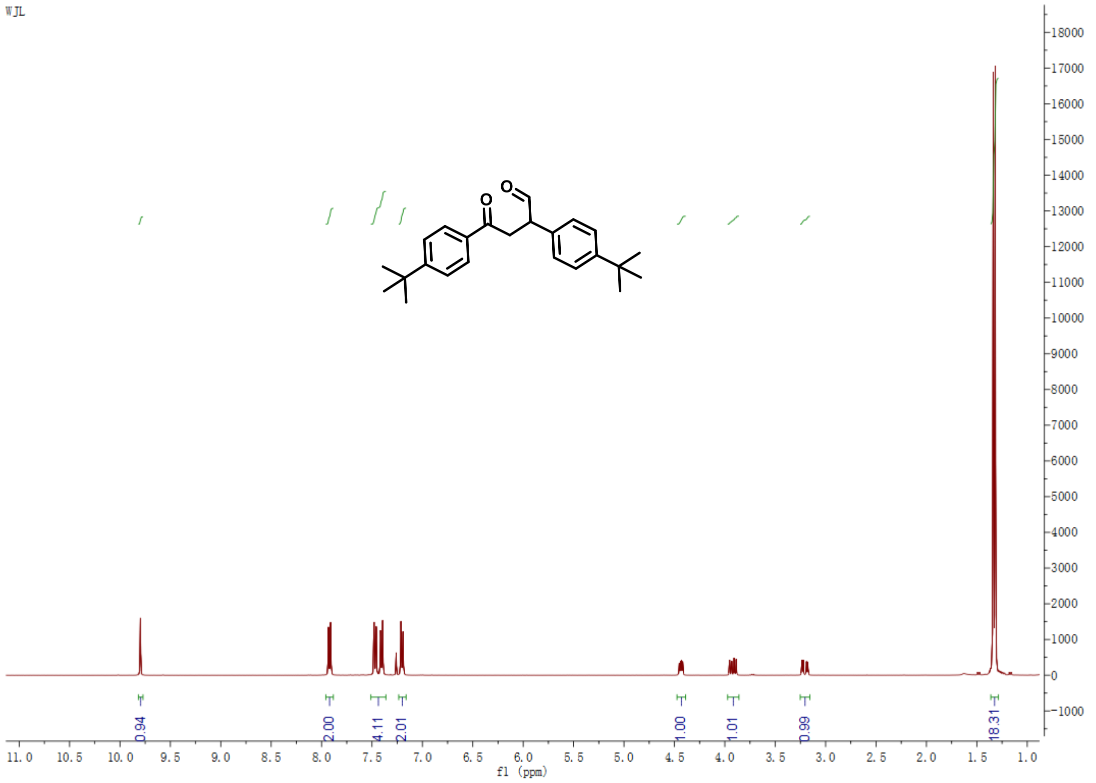

Figure S2. ^{1}H NMR spectrum of A2

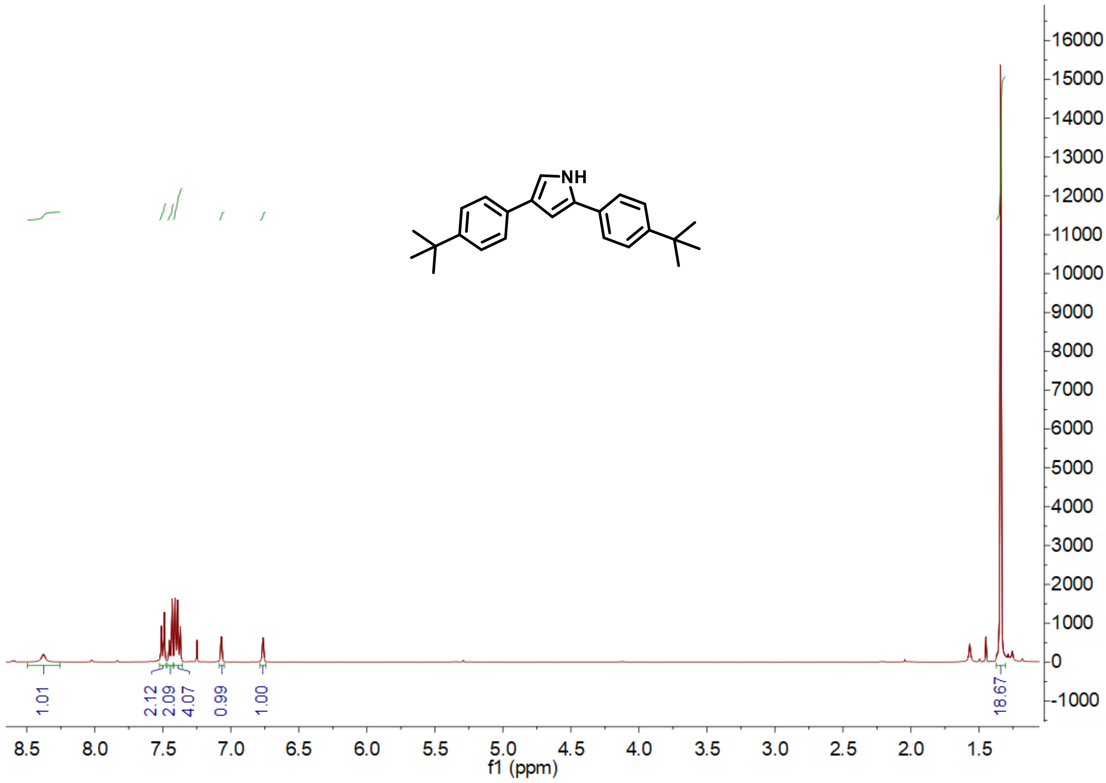

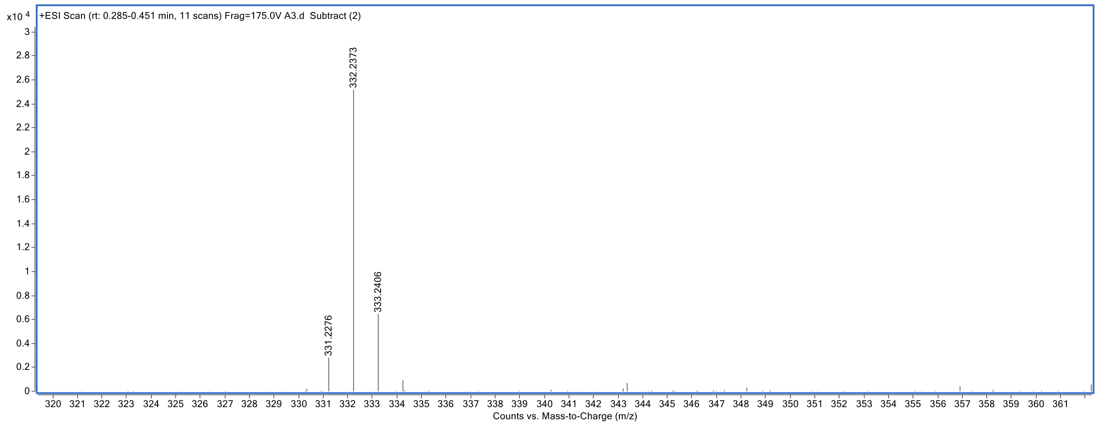

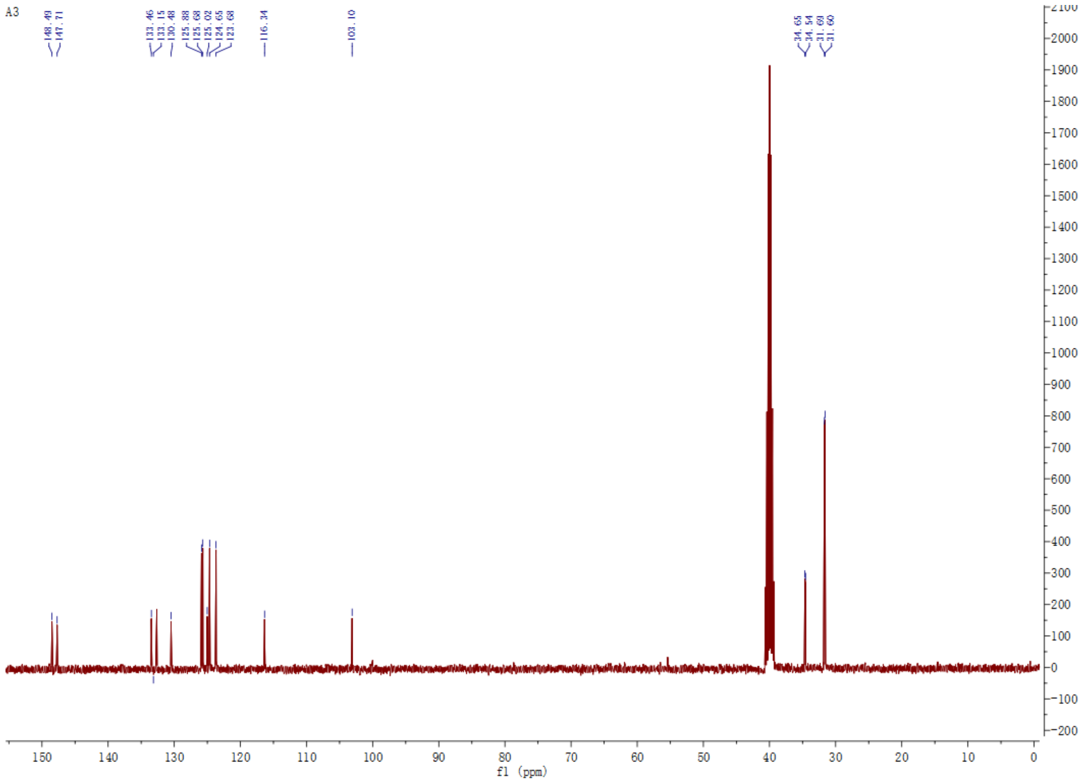

Figure S3. ^{1}H NMR spectrum (up), mass spectrometry (middle) and ^{13}C NMR spectrum (down) of A3.

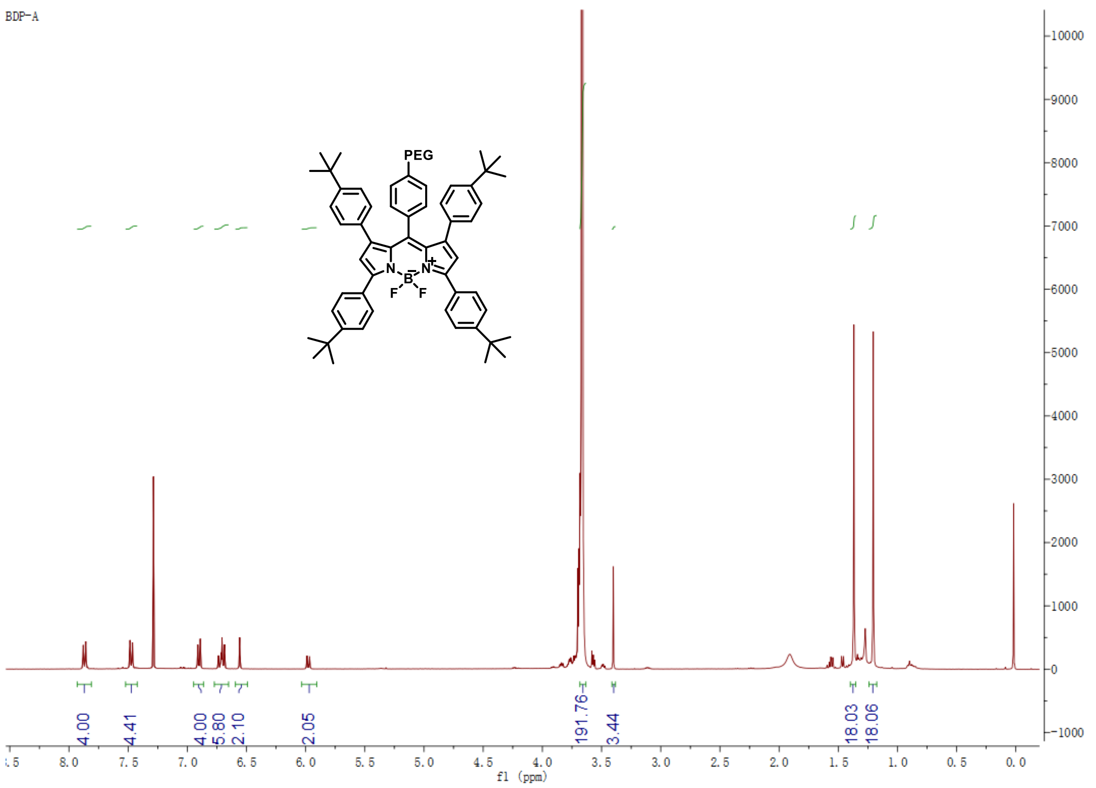

Figure S4. ^{1}H NMR spectrum of BDP-A

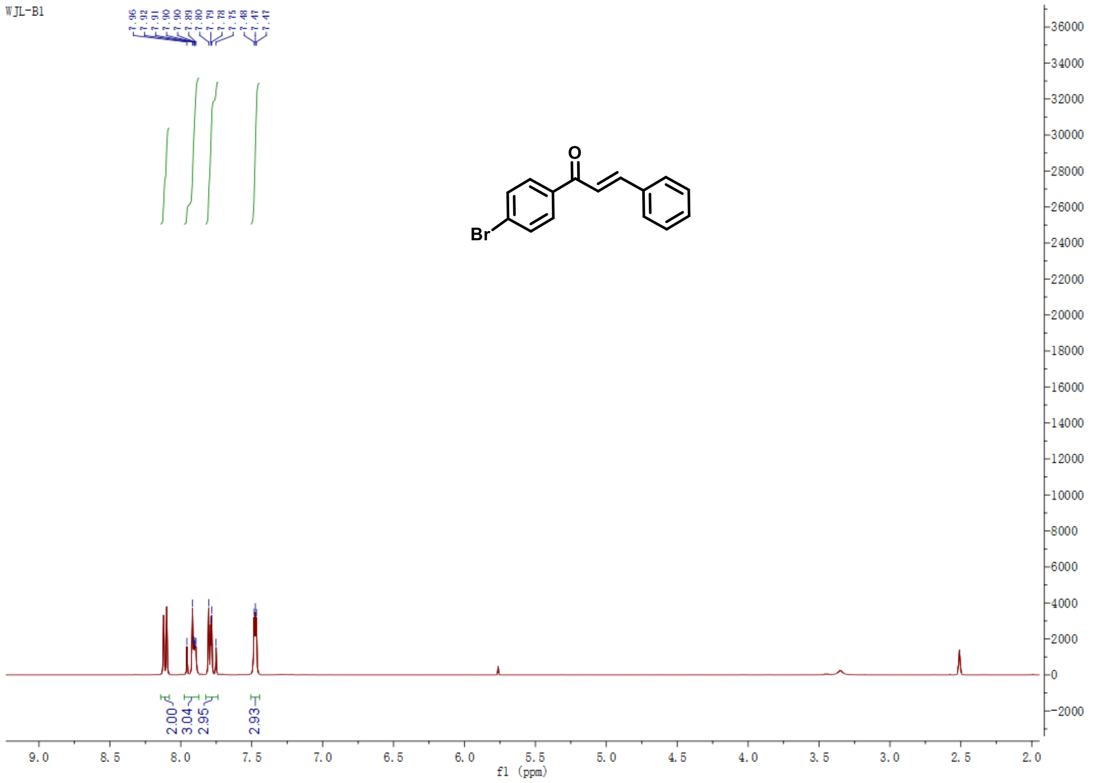

Figure S5. ^{1}H NMR spectrum of B1

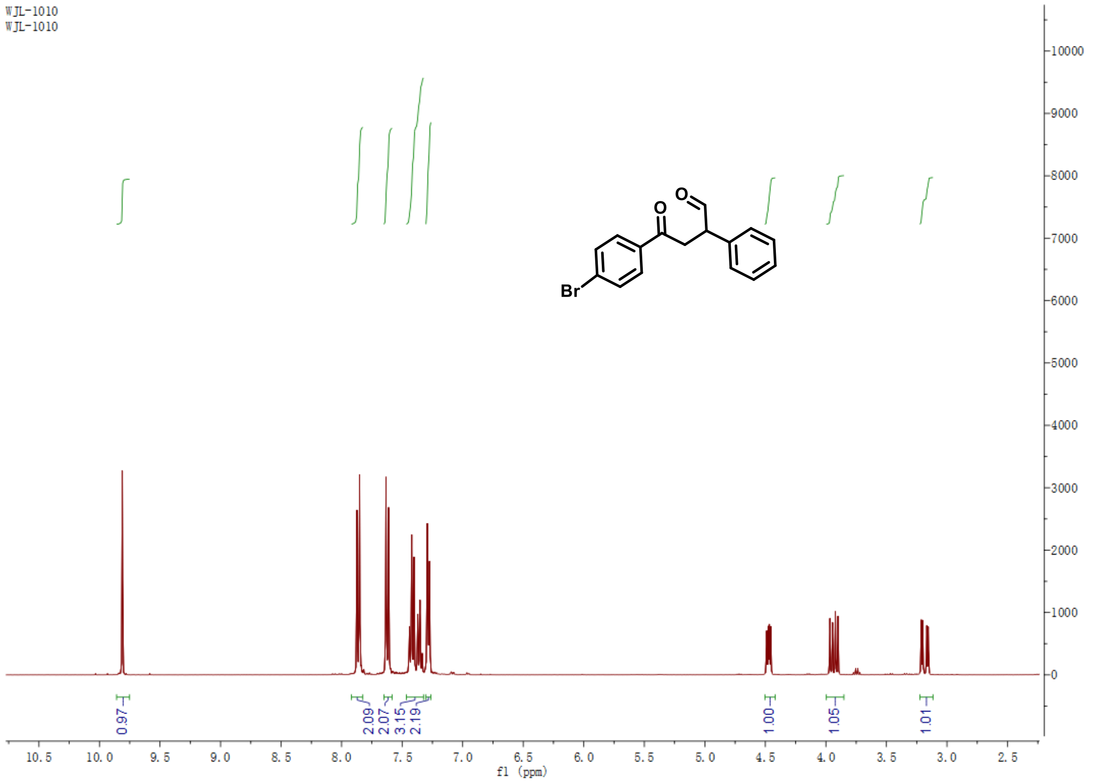

Figure S6. ^{1}H NMR spectrum of B2

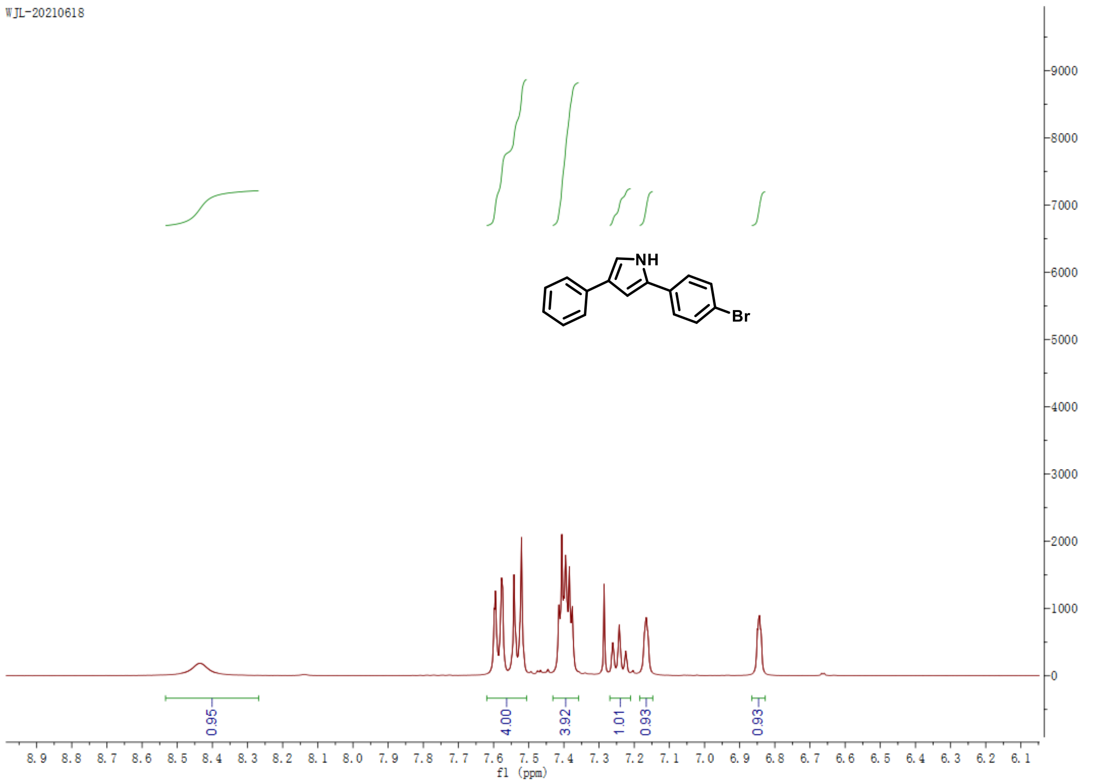

Figure S7. ^{1}H NMR spectrum of B3

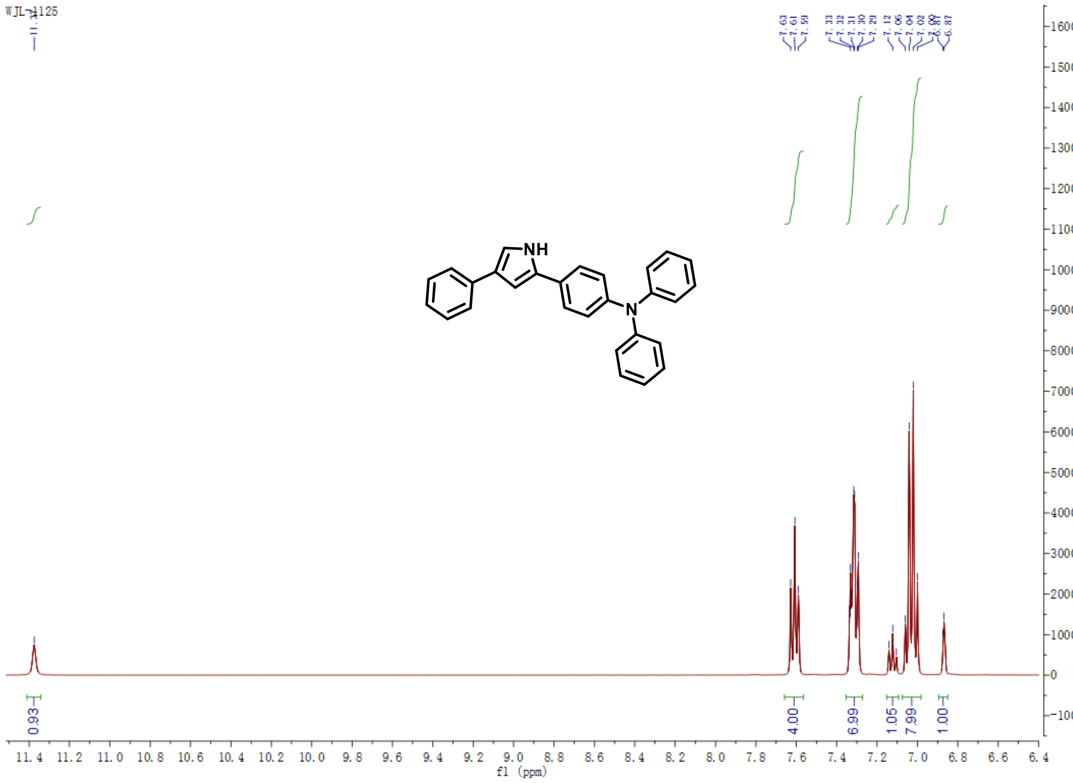

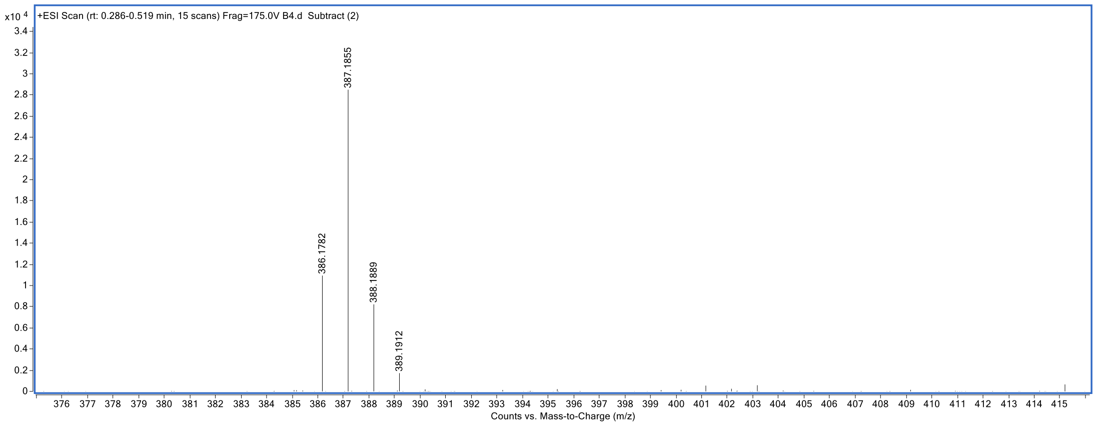

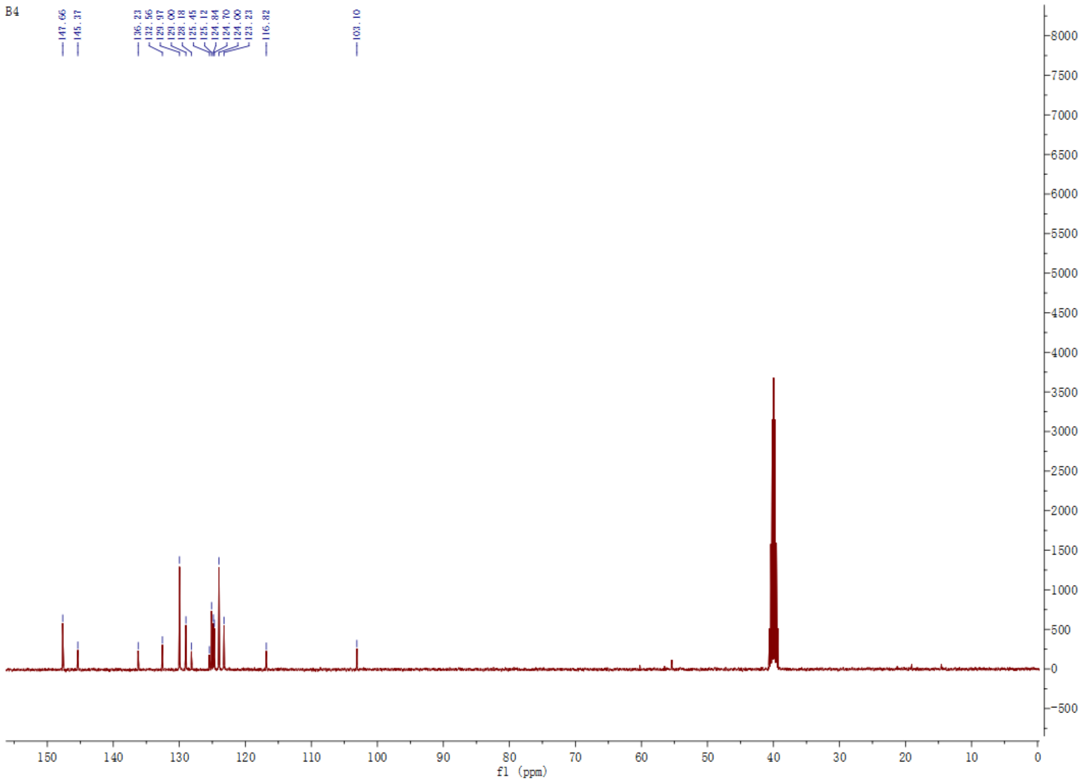

Figure S8. ^{1}H NMR spectrum (up), mass spectrometry (middle) and ^{13}C NMR spectrum (down) of B4.

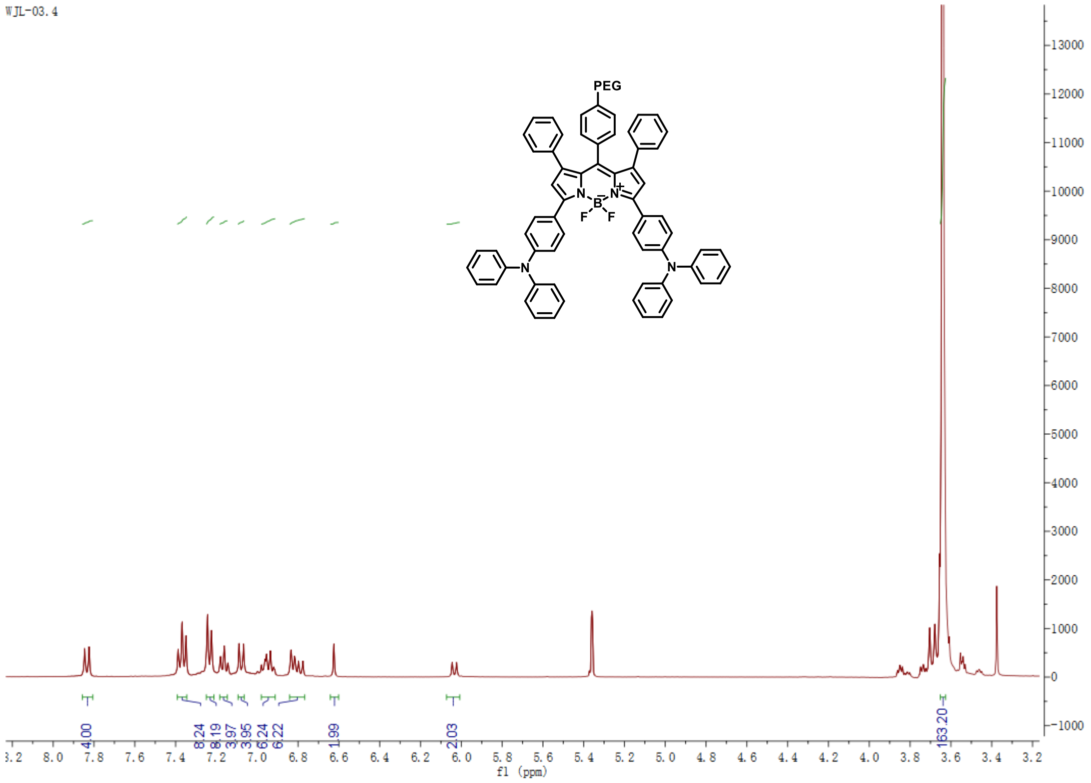

Figure S9. ^{1}H NMR spectrum of BDP-B

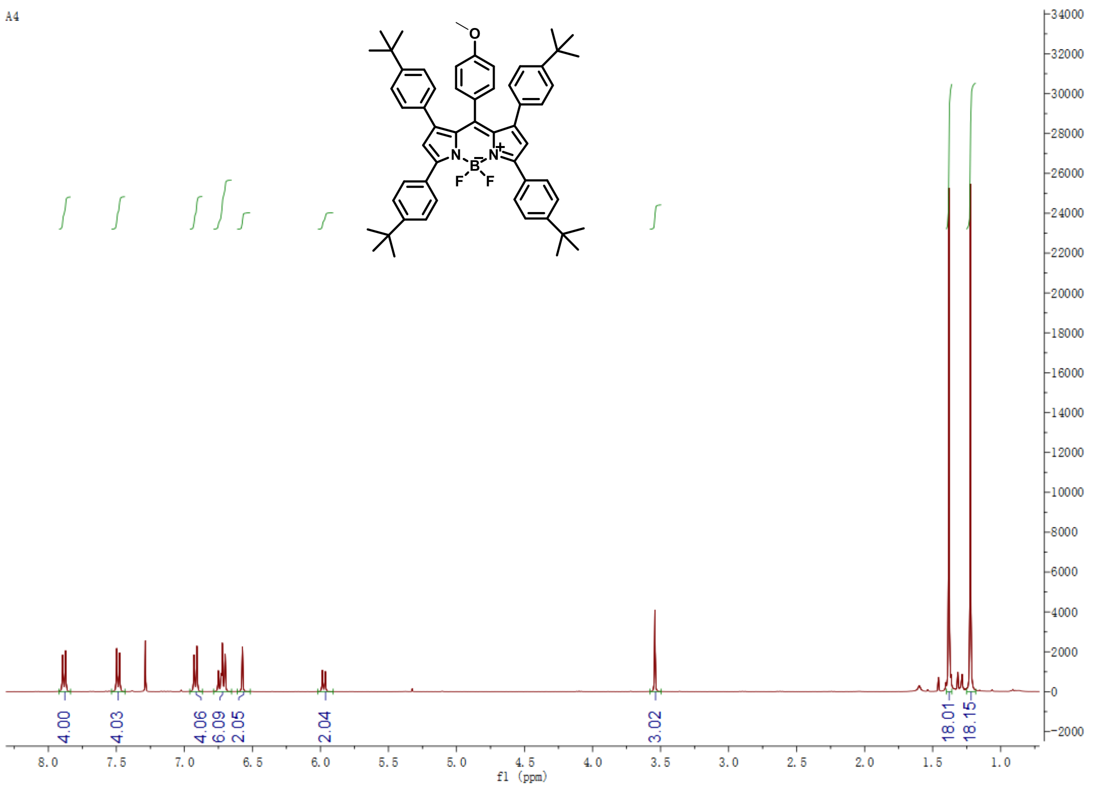

Figure S10. ^{1}H NMR spectrum of BDP-A with unattached PEG chains

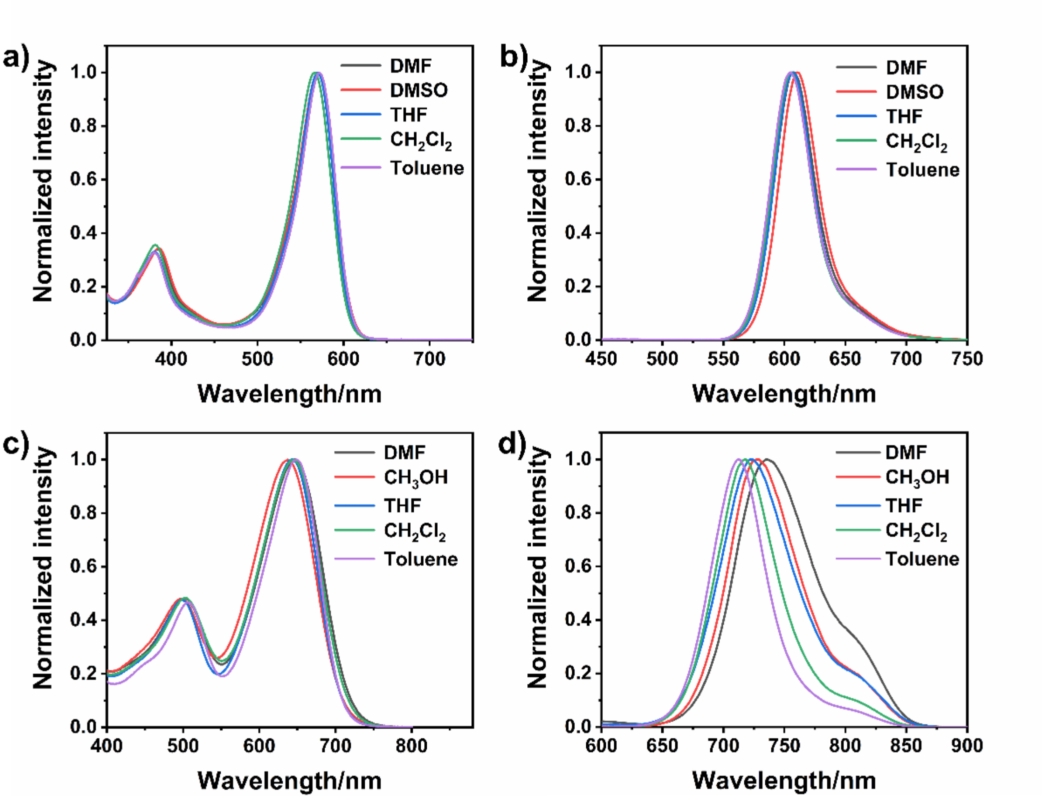

Figure S11. (a) Absorption spectra of BDP-A in different solvents. (b) Fluorescence spectra of BDP-A in different solvents. (c) Absorption spectra of BDP-B in different solvents. (d)

Fluorescence spectra of BDP-B in different solvents.

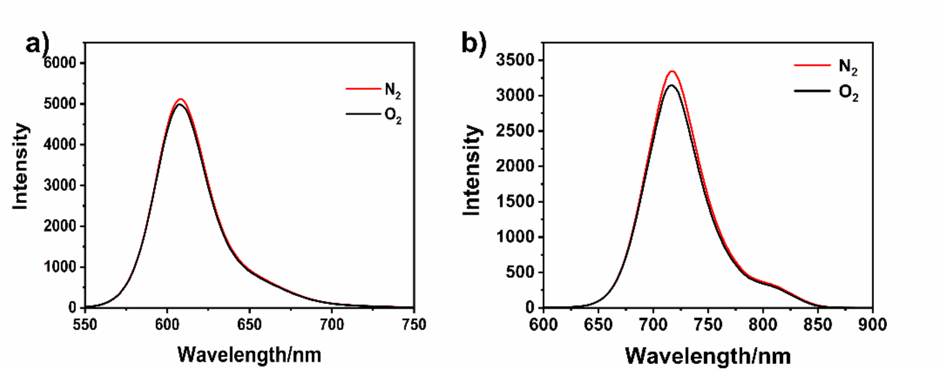

Figure S12. (a)The influence of oxygen on the fluorescence intensity of BDP-A (b)and BDP-B.

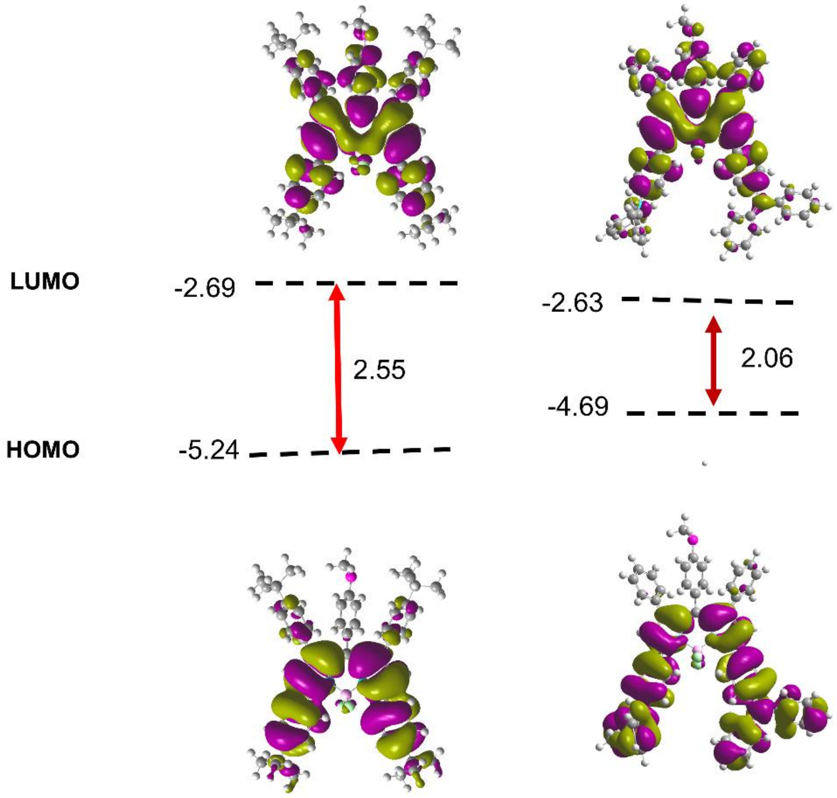

Figure S13. Theoretical calculation results of BDP-A and BDP-B

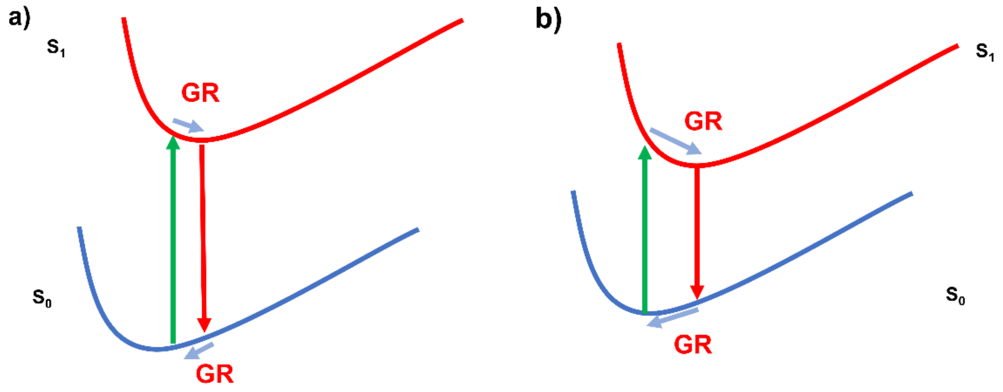

Figure S14. Simplified Jablonski diagram for the origin of the Stokes shifts of BDP-B. (a) Small geometry relaxation upon photoexcitation produces small Stokes shift, which is applicable to the unsubstituted BODIPY fluorophore. (b) Large geometry relaxation upon photoexcitation leads to large Stokes shift. GR stands for geometry relaxation.

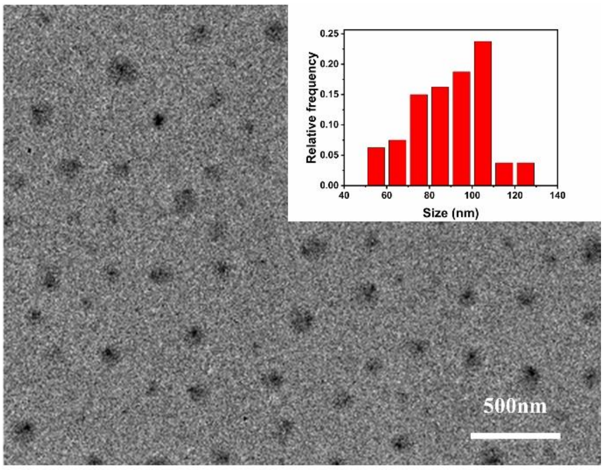

Figure S15. BDP-B particle size distribution and TEM imagine.

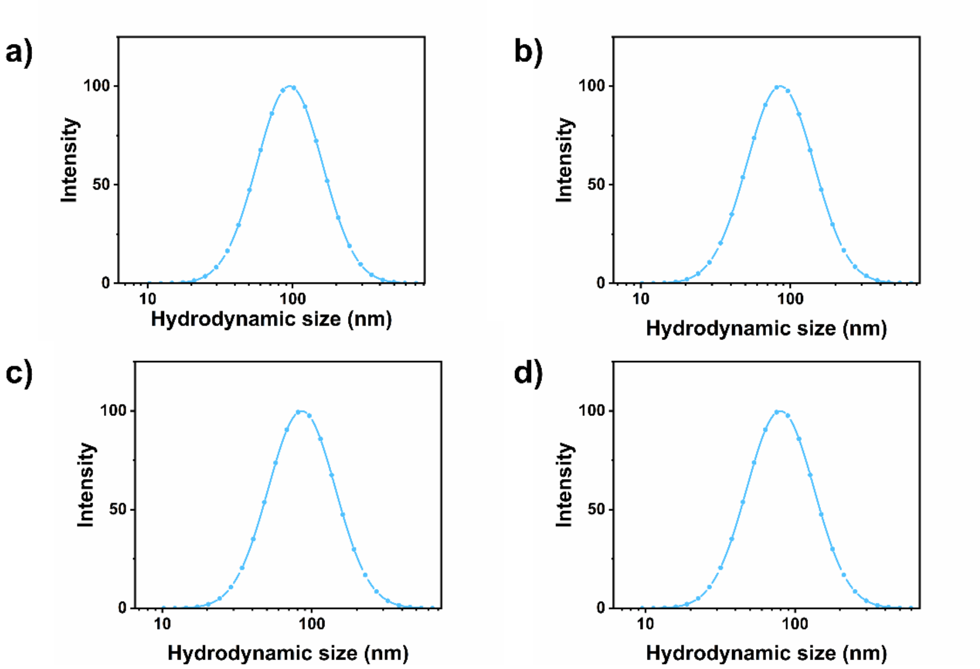

Figure S16. Intensity-weighted hydrodynamic size distribution plots over time at (a) 0h (b) 24h (c) 48h (d) 72h.

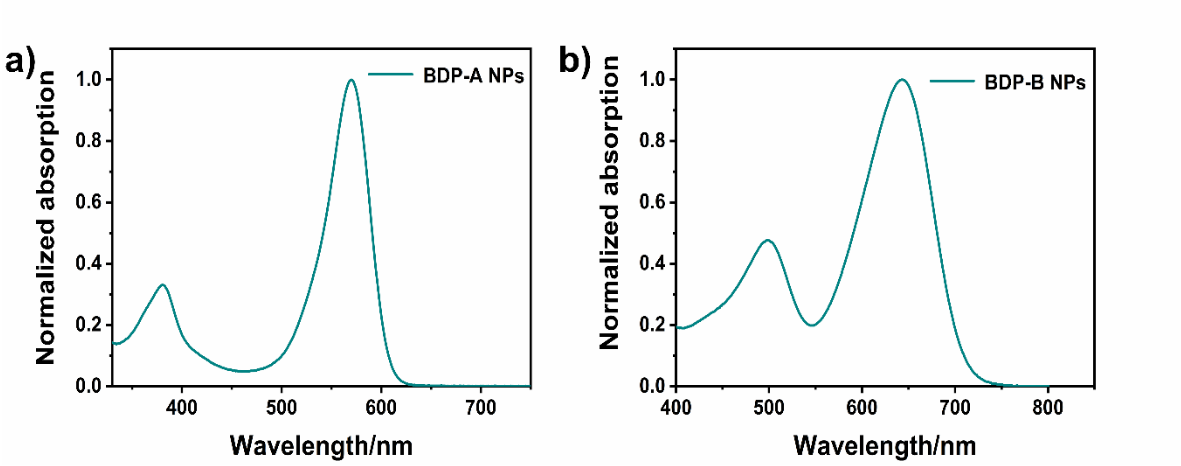

Figure S17. (a) Absorption spectra of BDP-A NPs and (b) BDP-B NPs.

Table S1. Crystallographic data of BDP-A.

	BDP-A
Empirical formula	$C_{56}H_{61}BF_2N_2O$
Formula weight	867.93
Temperature/K	296(2)
Crystal system	triclinic

Space group	P-1
a/Å	9.7765(5)
b/Å	15.3223(7)
c/Å	18.4821(9)
α/°	111.773(2)
β/°	99.359(2)
γ/°	93.807(2)
Volume/Å^3	2512.5(2)
Z	2
ρ_{calc}g/cm^3	1.147
μ/mm^{-1}	0.072
Radiation	MoKα (λ = 0.71073)
GOF	1.048
Reflections collected	24366
Independent reflections	11443 [Rint = 0.0384, Rsigma = 0.0620]
Data/restraints/parameters	11443/420/693
Final R indexes [I>=2σ (I)]	R_1 = 0.0625, wR_2 = 0.1693
Final R indexes [I>=2σ (I)]	R_1 = 0.1101, wR_2 = 0.1984

a)

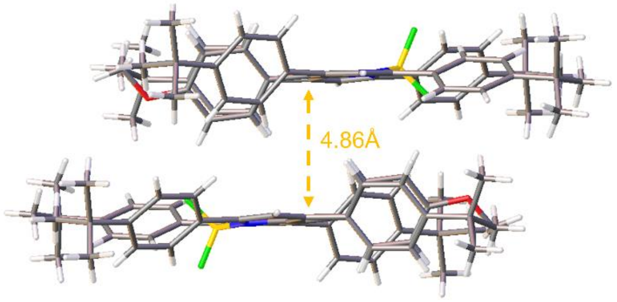

b)

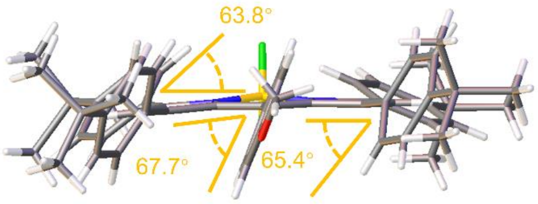

Figure S18. The dihedral angles between BODIPY core plane and meso-methoxybenzene or 1,7-tert-butyl benzene planes of BDP-A.

Table S2. Hemolytic test

Number	1	2	3	4	5	6	7
red cell suspension (mL)	2.5	2.5	2.5	2.5	2.5	2.5	2.5
normal saline (mL)	2.0	2.1	2.2	2.3	2.4	2.5	0
distilled water (mL)	0	0	0	0	0	0	2.5
BODIPY-A (mL)	0.5	0.4	0.3	0.2	0.1	0	0
BODIPY-B (mL)	0.5	04	0.3	0.2	0.1	0	0

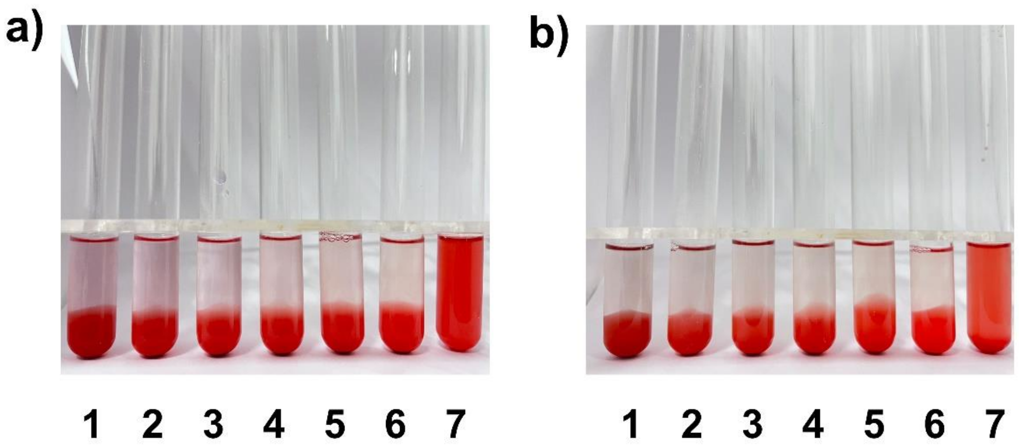

Figure S19. (a)Hemolytic rate of erythrocytes incubated with the BDP-A and (b) BDP-B at various concentrations.
